# Prevalence of *Giardia duodenalis* in dogs and cats: Age-related predisposition, symptomatic, and asymptomatic cyst shedding

**DOI:** 10.14202/vetworld.2024.379-383

**Published:** 2024-02-15

**Authors:** Olga P. Kurnosova, Olga A. Panova, Mikhail V. Arisov

**Affiliations:** Federal State Budget Scientific Institution, “Federal Scientific Centre VIEV” (FSC VIEV), 117218, Russia

**Keywords:** age of animals, cat feces, consistency of dog feces, coprological parameters, cysts, feces, *Giardia duodenalis*

## Abstract

**Background and Aim:**

Giardiasis is a protozoal disease that is globally prevalent in dogs and cats. The clinical manifestations vary, but asymptomatic cases have also been reported. This study aimed to estimate the prevalence of *Giardia duodenalis* in domestic dogs and cats, characterize the age susceptibility to this disease, and determine the frequency of cases in which cysts are combined with stool changes.

**Materials and Methods:**

After centrifugation in a zinc sulfate solution (specific gravity = 1.32 g/cm^3^), feces of dogs (2761) and cats (1579) were examined microscopically. The age of the animals and the presence of coprological stool characteristics such as fecal odor, consistency, presence of mucus, and other pathological impurities were taken into account.

**Results:**

*G. duodenalis* infection rates were 18.2% (215/1182) in dogs aged 1–12 months and 3.8% (60/1579) in dogs older than 12 months. The infection rate was 7.8% (48/615) in cats aged 1–12 months and 3.35% (33/994) in cats aged >12 months. The most frequently observed coprological abnormalities in cyst-positive animals were soft and/or mushy stool and pungent odor. In dogs, the frequency of these symptoms was 24.4% (67/275), 27.6% (76/275), and 36.4% (100/275) for soft stools, mushy stools, and pungent fecal odor, and 37.8% (31/82), 25.6% (21/82), and 19.5% (16/82), respectively. No stool changes were found when *G. duodenalis* cysts were detected in dogs in 24.7% (68/275) of cases and in cats in 24.4% (20/82).

**Conclusion:**

*G. duodenalis* cysts are reported more frequently in domestic cats and dogs under 12 months of age than in dogs under 12 months of age. The presence of *Giardia* cysts is usually accompanied by a strong odor of feces and changes in their consistency. It can be concluded that it is necessary to conduct periodic surveillance for apparently healthy dogs and cats to rule out *G. duodenalis* infection.

## Introduction

Giardiasis is a parasitic disease caused by flagellate protozoans [1, 2]. In both developed and developing countries, these protozoa frequently cause acute gastroenteritis in humans and many animal species worldwide [3–5]. *Giardia duodenalis* parasitizes the small intestine and disrupts parietal digestion [4]. The clinical picture is diverse, with dyspeptic manifestations in the form of diarrhea, abdominal pain, anorexia, and weight loss being the most characteristic signs [4, 6]. Factors such as the state of the immune system, *G. duodenalis* genotype, age of the animal, and the presence of concomitant gastrointestinal diseases may also influence the manifestation of the disease [2, 4, 7–11]. In spite of its worldwide distribution, the problem of giardiasis is most acute in hot countries, where the prevalence of giardiasis depends on sociocultural and sanitary conditions [9, 12–15]. The risk group includes children with poor personal hygiene skills, adults whose occupation conditions are associated with the infestation and people with immunodeficiency diseases [6, 9, 12, 16].

In most cases, dogs and cats are infected with species-specific isolates of *G. duodenalis*; however, genotypes A and B, which are common to humans [3, 4, 17–19], are also present. Close contact with family members is likely when dogs and cats are housed [6, 10]. Therefore, animal-to-human transmission of the infestation is an urgent and most discussed issue in the academic community [3, 4, 9, 17–23]. In large cities, there are many domestic dogs, and it is common for animals to be walked in limited areas that may overlap with stray animal habitats [9]. Fecal contamination in these areas can be significant, particularly in spring, which may be a favorable factor for *G. duodenalis* infection [9, 16]. Crowding in shelters or kennels favors *Giardia* infection [14, 24]. All of these factors lead to a significant spread of giardiasis and other infections that can be transmitted to humans [16, 17].

More recent studies on the prevalence of *G. duodenalis* in Moscow did not focus on the age of the animals and the presence of coprological abnormalities in their feces. This study aimed to estimate the prevalence of *G. duodenalis* in domestic dogs and cats, characterize the age susceptibility to this disease, and determine the frequency of cases in which cysts are combined with stool changes.

## Materials and Methods

### Ethical approval and Informed consent

The study protocol was reviewed and approved by the scientific and methodological commission of Federal State Budget Scientific Institution, “Federal Scientific Centre VIEV” (FSC VIEV) (Protocol No. 1 dated 08/02/2018). Written informed consent was obtained from the animal owners for the participation of their animals in this study.

### Study period and location

This study was conducted from May 2018 to December 2022 in a Veterinary Laboratory in Moscow (Russia).

### Collection and examination of fecal samples

Feces of asymptomatic animals or animals with diarrhea were delivered to the laboratory by the owners or by courier services from different veterinary doctors of Moscow. The following parameters were evaluated during the study: Age of the animal and coprological characteristics of feces at the time of the study: Odor, consistency, presence of mucus, and visible blood in the stool. All samples were delivered and labeled in plastic packaging. The feces were examined using the flotation method with centrifugation using zinc sulfate (ZnSO_4_) solution with a density of 1.24. First, the feces were washed with water. For this purpose, feces (2–5 g) were mixed with water in plastic cups, filtered through two layers of gauze into a 15-mL centrifuge tube, and centrifuged at 650× *g* for 15 min. The tube contents were drained, ZnSO_4_ solution was added, and centrifugation was repeated. The centrifuge was stopped after 5 min, a drop was transferred from the surface of the liquid layer to a slide using a bacteriological loop, and microscopy was performed without using a slide [25].

Microscopic examination was performed using a Lomo microscope (Joint-stock company Lomo, Russia) at magnifications of 10× and 40×.

### Statistical analysis

To determine the dependence of the degree of infestation on age in months, the analysis was based on statistical methods such as the Chi-square test of independence of distributions and pairwise Z-tests with Bonferroni correction for multiple comparisons. All comparisons had a p-value of 0.05. Data were analyzed using IBM SPSS Statistics version 26.0 (IBM SPSS, NY, USA).

## Results

A total of 2761 dog feces samples and 1579 cat feces samples submitted to the city laboratory were examined. A comparative analysis of the prevalence of infestation in cats and dogs under 12 months of age and over 1 year of age allowed us to confirm the hypothesis that the extent of infestation in older animals was statistically significantly lower (χ^2^[1] = 15.985, p < 0.001 for cats and χ^2^[1] = 156.074, p < 0.001 for dogs). The rates of infestation extensiveness in domestic dogs and cats under 12 months of age were significantly higher (18.2%/1182) than those in animals older than 12 months of age (3.8%/1579), 7.8%/615 in cats under 12 months of age and 3.3%/994 in cats older than 12 months of age (Table-1).

**Table-1:** Prevalence of *G. duodenalis* infection by age group and animal species.

Type of animals	Giardia spp.	Total		1–12 months		More than 12 months		Chi-square/p-value
		
		Quantity	%	Quantity	%	Quantity	%	
Cats	Not detected	1528	95.0	567	92.2	961	96.7	15.985
	Detected	81	5.0	48	7.8	33	3.3	<0.001
	Total	1609	100.0	615	100.0	994	100.0	
Dogs	Not detected	2486	90.0	967	81.8	1519	96.2	156.074
	Detected	275	10.0	215	18.2	60	3.8	<0.001
	Total	2761	100.0	1182	100.0	1579	100.0	

*G. duodenalis=Giardia duodenalis*

**Figure-1:**
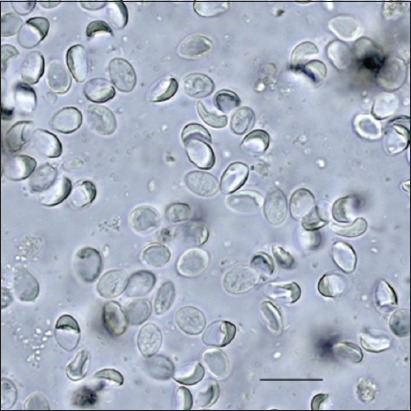
Cysts of *Giardia duodenalis*. Scale bar 10 μm.

**Figure-2:**
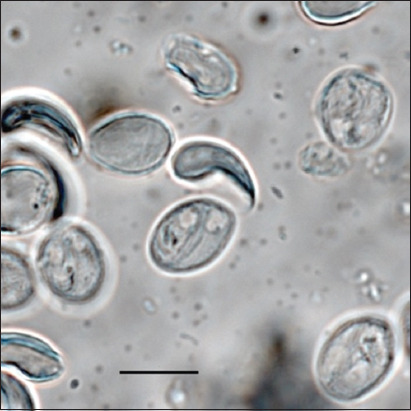
Cysts of *Giardia duodenalis*. Scale bar 20 μm.

**Figure-3:**
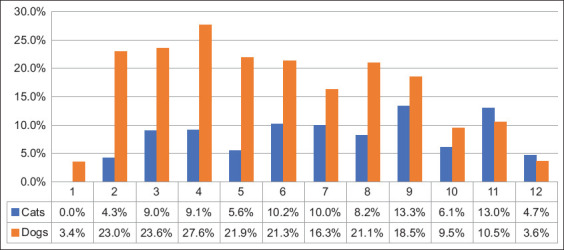
Prevalence of *Giardia duodenalis* infection among dogs and cats by age.

For a more detailed analysis of the relationship between the degree of infestation and the age of dogs and cats, monthly data were analyzed for the first year of life (Figures-1 and 2). A Chi-square test of independence showed no statistically significant relationship between age and the prevalence of infestation in cats (χ^2^[11] = 8.306, p = 0.686); however, this relationship was found in dogs (χ^2^[11] = 45.569, p < 0.001). A more detailed pairwise comparison of the prevalence of infestation in dogs, carried out at the monthly level, showed that at 12 months of age, the lowest rate of 3.6% was statistically significantly lower than that at 2–9 months, with a prevalence of 18.5%–23% and a maximum of 27.6% at 4 months of age. Only one case of infection was detected in newborn animals aged 1 month, which makes the comparative analysis for this group incorrect due to the ultra-small set of cases (Figure-3).

The main changes in coprological parameters when *G. duodenalis* cysts were detected were recorded in this study. The most common symptoms in cats were soft stools (37.8%), mushy stools (25.6%), and pungent odor (19.5%). For dogs, the same changes were noted, but in a different order of frequency: pungent odor (36.4%), mushy stool (27.6%), and soft stool (24.4%). Combinations of several coprological abnormalities have also been observed in cats and dogs; however, due to their rarity, these combinations were not subjected to statistical analysis. *G. duodenalis* cyst excretion without changes in coprological parameters was observed in 24.4% of cats and 24.7% of dogs (Table-2).

**Table-2:** Carpological parameters in animals with *G. duodenalis* infection.

Symptoms	Type of animals			

	Cats		Dogs	
	
	Frequency	%	Frequency	%
No change	20	24.4	68	24.7
Pungent odor	16	19.5	100	36.4
Soft stool	31	37.8	67	24.4
Diarrhea	4	4.9	10	3.6
Mushy stool	21	25.6	76	27.6
Sour odor	4	4.9	1	0.4
Mucous stool	0	0.0	5	1.8
Fetid odor	0	0.0	1	0.4
Total	82	100.0	275	100.0

*G. duodenalis=Giardia duodenalis*

## Discussion

Our results showed patterns of *G. duodenalis* prevalence in the pet population comparable to those reported in other studies. However, prevalence rates vary and depend on many factors, such as climate, living conditions of the animal, age, method of investigation, and groups of animals studied [3, 26–31]. *Giardia* is detected more frequently than other intestinal parasites in many regions. The age of an animal is considered to be a risk factor for Giardia infestation [2, 30–33]. In our study, the infestation rate was 18.2% in dogs under 12 months of age and 7.8% in cats. Sweet *et al*. [30, 34] reported that *Giardia* was found in 12.2% of cases in puppies aged 2–6 months of age, and a similar trend was observed in cats, regardless of the method of study. Gultekin *et al*. [32] reported that *G. duodenalis* was prevalent in puppies in up to 18.8% of the cases. Abdulaziz and Sorour [35] reported a prevalence of up to 9.5% in puppies less than 6 months of age. Piekara-Stępinska *et al*. [29] reported a prevalence of up to 12.2% in *G. duodenalis* cysts in dogs under 12 months of age. Murnik *et al*. [28] reported a prevalence of up to 29% in *Giardia* cysts in dogs under 12 months of age. The highest prevalence of *Giardia* spp. was observed in dogs living in crowded housing. Agresti *et al*. [26] reported a prevalence of *G. duodenalis* cysts in 72.7% of pups under 6 months of age in shelters. Adam [1] attributes the susceptibility of young animals to *Giardia* infection to good parietal digestion, which is a prerequisite for successful colonization of the small intestine. In addition, the high susceptibility of young animals is also due to the lack of immunity to this infection, which develops with age and provides immunity to re-infection [36].

One of the mechanisms of giardiasis pathogenesis is impaired intestinal barrier function, which leads to the clinical manifestation of infection [36]. The clinical manifestations of giardiasis may be acute, chronic, or asymptomatic. Clinical symptoms include diarrhea, vomiting, weight loss, abdominal pain, and dehydration. In addition to the protozoa, the manifestation of infestation is influenced by the state of the immune system, age, dietary peculiarities, pathogen genotype, dose of infection, and possible concomitant infections [27]. Our study has shown that the excretion of *G. duodenalis* cysts is most often accompanied by a pungent fecal odor in 36.4% of cases and a mushy consistency of feces in 27.6% of cases in dogs and 19.5% and 37.8% of cases in cats, respectively. In the presence of giardia, 24.7% of cases in dogs and 24.4% of cases in cats did not change the stool characteristics.

The number of asymptomatic and symptomatic animals varies according to the reports of different researchers. For example, Li *et al*. [33] found that 11.8% of dogs with giardiasis have diarrhea, Uiterwijk *et al*. [2] reported that in 28.9% of cases, *Giardia* spp. cysts were found without changes in stool pattern, Claerebout *et al*. [37] reported the presence of symptoms and *G. duodenalis* isolation in dogs in 18.1% of cases. Gultekin [32] identified 24.6% of dogs with diarrhea whereas 10.2% of them had no symptoms, Abdulaziz and Sorour [35] identified diarrhea in 14.4% of dogs. Piekara-Stępinska *et al*. [29] and Murnik *et al*. [28] showed no statistically significant differences related to fecal consistency and excretion of *G. duodenalis* cysts in the studied groups of animals. We believe that the frequent presence of *G. duodenalis* cysts and the absence of coprological abnormalities in our study may be due to a single examination. We did not exclude the possibility of detecting changes in stool patterns during repeated examinations in these animals.

Because dogs and cats excrete genotypes common to humans, there is a risk of zoonotic transmission, particularly in asymptomatic infections, when carrier animals may excrete cysts into the external environment for a considerable time [13, 27, 28, 38–40]. Therefore, routine testing for *G. duodenalis* infection in different habitats is of great importance [30]. This is particularly important for young animals at risk of giardiasis. In addition, it allows the identification of animals without symptoms. In addition to the timely treatment of giardiasis in clinical and asymptomatic animals, these studies will help to assess the current prevalence of these protozoa and develop timely preventive measures.

## Conclusion

The findings of this study suggest that a high prevalence of *G. duodenalis* was observed among pets in Moscow. Animals under 12 months of age are the most vulnerable. The release of giardia cysts is more often accompanied by coprological abnormalities, but asymptomatic cyst release has also been reported. The obtained data make it possible to conclude that the feces of animals under 12 months of age must be periodically monitored, even in the absence of giardiasis symptoms.

## Authors’ Contributions

OPK: Conceived and designed the study. OPK and OAP: Collected the samples and data analysis. OPK, OAP, and MVA: Interpreted the data, and drafted the manuscript. All authors have read, reviewed, and approved the final manuscript.
